# Minor Ailments in Pregnancy Are Not a Minor Concern for Pregnant Women: A Morbidity Assessment Survey in Rural Sri Lanka

**DOI:** 10.1371/journal.pone.0064214

**Published:** 2013-05-10

**Authors:** Suneth Buddhika Agampodi, Nuwan Dharshana Wickramasinghe, Jennifer Horton, Thilini Chanchala Agampodi

**Affiliations:** Maternal and Child Health Research Unit, Department of Community Medicine, Faculty of Medicine and Allied Sciences, Rajarata University of Sri Lanka, Saliya pura, Sri Lanka; Tabriz University of Medical Sciences, Iran (Republic of Islamic)

## Abstract

**Background:**

Although maternal mortality has become a major focus on global public health agenda, maternal morbidity is a neglected area of research. The purpose of this paper is to present the burden of acute maternal illness during pregnancy.

**Methods:**

A cross sectional study was carried out in Anuradhapura district, Sri Lanka. Pregnant women residing in the Anuradhapura district with a gestational age more than 24 weeks through 36 weeks were recruited to the study using a two-stage cluster sampling technique. All pregnant women who consented participated in a detailed interview using a structured questionnaire. Self reported episodes of acute illness during pregnancy were the main outcome measures. Secondary outcomes were utilization of medical services and frequency of hospitalizations.

**Results:**

Nausea and vomiting during pregnancy (NVP) was experienced by 325 (69.7%) of the 466 pregnant women studied. Other common symptoms were backache (152, 32.6%), dizziness (112, 24.0%) and heartburn/regurgitation (107, 23.0%). Of the 421 pregnant women who reported ill health conditions 260 (61.8%) women sought medical treatment for these illnesses. Total number of episodes that needed treatment seeking were 373. Hospitalizations were reported by 83 (17.8%) pregnant women and the total number of hospitalizations was 109. The leading cause of hospitalization was NVP which accounted for 43.1% of total admissions and 49.1% of total days spent in hospitals.

**Conclusions:**

Minor maternal ill health conditions affecting day-to-day life have a major burden on pregnancy period. Evidence based management guidelines and health promotion strategies are needed to control and prevent these conditions, in order to provide comprehensive, good quality maternal health care.

## Introduction

In 2007, evidence synthesis in the background paper for “Women Deliver” argues that “maternal health is connected with women’s lives and options as individuals, the well-being of their children and families, and the economic productivity of their countries” [1]. The well being of pregnant women has continued to be threatened, despite global initiatives aimed towards reduction of maternal mortality. Global estimates on maternal deaths shows around 287,000 maternal deaths in 2010, a 47% reduction compared to 1990 figure of 540,000 deaths [2]. In addition to maternal mortality, severe maternal morbidities impact up to 9% of pregnant women [3]. The central focus of the global maternal health agenda is on maternal mortality, whereas the impact of less severe morbidities accounting for an estimated 58–80% of acute ill conditions [1] affecting pregnant women in developing countries are often overlooked.

Studies on minor acute illness during pregnancy suggests that despite being non-life threatening, the high prevalence of these conditions has a major effect on productivity and may have profound impact on the lives of pregnant women and their families [4], [5]. Yet, surveillance of so-called “minor ailments” during pregnancy is virtually nonexistent in developing countries,This lack of reliable data impedes proper assessment of the disease burden and is a barrier to effective planning of control and preventive activities.

The Sri Lankan case study is often used in global maternal health agenda as a cost effective and highly successful programme which reduced maternal mortality by 50% within 10–12 years [1]. A well-structured maternal mortality surveillance system, a combined system of community based verbal autopsy and institutional based confidential inquiry in Sri Lanka has been an essential component of this successful programme. However, maternal morbidity surveillance in Sri Lanka has yet to be developed. Recent studies on morbidity surveillance have shown some of the quality and coverage issues of the present surveillance system [6], [7]. Though national policy and guidelines are in place, first-hand reports suggest that morbidity surveillance in the field may suffer both from poor resource allocation by the government and poor commitment of the field staff, secondary to a lack of prioritization. One major reason for the lack of prioritization is the scarcity of data on actual disease burden.

The Department of Community Medicine, Faculty of Medicine and Allied Sciences, Rajarata University of Sri Lanka initiated the project titled “The disease burden and economic impact of maternal morbidities in Sri Lanka” with collaboration of Maternal Health Task Force at EngenderHealth to fill knowledge gaps about maternal morbidity in the Anuradhapura province. The main purpose of the project was to estimate the community prevalence of maternal ill health conditions and their economic impact [8]. In 2012, we showed that the minor ailments were the leading causes of productivity cost among Sri Lankan pregnant women [9]. The purpose of the present paper is to enumerate and discuss the burden of acute ill health among pregnant women, paying special attention to minor ailments during pregnancy.

## Methods

### Ethics Statement

Ethical approval for this study was obtained under the larger project “ Disease burden and economic impact of maternal morbidity in Sri Lanka” from the ethical review committee of Faculty of Medicine and Allied Sciences, Rajarata University of Sri Lanka. Written informed consent was obtained from all participants. Pregnant women who were having conditions that needed urgent medical attention, but not on treatment were referred to tertiary care center through area MOH.

### Design

We carried out a community-based study in the Anuradhapura district, Sri Lanka, with a primary goal of determining the common symptoms of illness during pregnancy. This study was performed as one arm of a larger study on productivity cost of maternal morbidity among pregnant women which was done using the same study population and published in 2012 [9].

### Study Sample and Sampling Procedure

In the maternal morbidity study, we aimed at the self-reported episodes ill health excluding the major perinatal conditions in and around labor. The study population included pregnant women residing in Anuradhapura district with a gestational age more than 24 weeks, up toan 36 weeks. Pregnant women with a gestational age more than 36 weeks were not included to avoid major perinatal conditions in this study. Sample size was calculated to detect at least 70% of acute ill conditions during pregnancy within 95% confidence limits and.05 precision. A two-stage cluster sampling procedure was carried out to select the study sample. In the first stage, four Medical Officer of Health (MOH) areas were selected from Anuradhapura district. In the public health system of Sri Lanka, each MOH area is subdivided into multiple Public Health Midwife (PHM) populations of one PHM per 3,000 general population. In the second stage we invited all pregnant women between gestational ages 24–36 weeks who were registered in the pregnant mother register to a PHM to participate in the present study. Informed consent was sought from all participants. The registration of pregnant mothers to their local PHM is 100%, based upon the quarterly maternal and child health returns of the four areas. Thus we assumed this sampling procedure reached the whole study population.

### Data Collector and Data Collection

Data collectors were trained and protocols were developed for data collection, as previously described [9]. Each possible symptom was previously identified through a literature review and a series of focus group discussions. The focus group discussions were carried out to identify different terms used by local women to describe common antepartum ill health conditions. In total, four focus groups with pregnant woman in the Anuradhapura district were held in addition to one focus group with local PHMs. Data on common antepartum symptom terminology was consolidated and informed development of the structured interview template. Symptoms were predefined for interview purposes to minimize interviewer variability. Detailed questions regarding symptoms and their impact were included in the protocols. Secondary reports were used on site to extract data on morbidities and follow-ups were done for missing data, through area PHMs.

All consented pregnant women underwent a detailed interview using the structured questionnaire. Data on the symptoms, frequencies, treatment, hospitalization, and effect on day-to-day life in the antepartum period were collected. Subjects were asked to recall any episodes of ill health over the course of their pregnancy, as defined by the questionnaire protocol. Hospitalizations treatments received were confirmed using diagnosis cards and medical records. Effect on day-to-day activity was measured using a visual analog scale of 0 to 5; 0 representing total incapacitation due to illness and five representing no effect. This visual analog scale was adopted from the validated Sinhalese translation of culturally adapted IMMPACT productivity cost questionnaire, which was validated using concurrent and convergent validity [9].

To minimize recall bias, which is an inherent challenge to cross-sectional studies, we used available medical records, notes in antenatal chart and clinic books as further confirmation of episodes of illness for each woman interviewed.

## Results

Of the 502 women who were eligible as study units and were invited to participate, 484 responded and 466 participants provided complete data for the present analysis. Mean age of the study sample was 27.3 years (SD 5.5 years). Sample consisted mainly of Sinhalese women (93.8%), aged 20–35 years (82.8%) in the early part of third trimester (Table 1).

**Table 1:** Characteristics of the study sample.

	Count	%
**Age**		
<20 years	36	7.7%
20–35 years	386	82.8%
>35 years	44	9.4%
**Ethnicity**		
Sinhalese	437	93.8%
Tamil	3	.6%
Moor	26	5.6%
**Gestational age**		
24–28 weeks	156	33.5%
29–36 weeks	309	66.5%
**Parity**		
1	191	41.0%
2	141	30.3%
3 or more	134	28.8%
**Highest level of education**		
Up to grade 5	17	3.7%
Up to grade 10	212	45.7%
Passed O/L	128	27.6%
Passed A/L	83	17.9%
University education	24	5.2%

Of the 466 pregnant women studied 421 (90.3%) reported at least one episode of ill health during pregnancy. One hundred (21.5%) women reported one episode of ill health, 80 (17.2%) reported two episodes. The remaining women (57.2%) reported three or more different types of illness. Nausea and vomiting during pregnancy (NVP) was experienced by 325 (69.7%) of the study sample (Table 2). Other common symptoms experienced by the study samples were backache (152, 32.6%), dizziness (112, 24.0%) and heartburn/regurgitation (107, 23.0%).

**Table 2:** Symptoms of acute ill health conditions and effects of ill health among 466 pregnant women gestational age 24–36 weeks in Anuradhapura, Sri Lanka.

Symptom	Number experienced (N = 466)	%	Perceived effect on daily activities					
			No effect	%	Mild to moderate	%	Severe/total incapacitation	
**Nausea and vomiting**	325	69.7	53	16.3	168	51.7	104	32.0
**Backache**	152	32.6	49	32.2	77	50.7	26	17.1
**Dizziness**	112	24.0	29	25.9	60	53.6	23	20.5
**Heartburn/regurgitation**	107	23.0	31	29.0	60	56.1	16	15.0
**Cramps**	102	21.9	45	44.1	46	45.1	11	10.8
**Pain in pelvic area**	95	20.4	31	32.6	37	38.9	27	28.4
**Cough and cold**	83	17.8	36	43.4	40	48.2	7	8.4
**Headache**	76	16.3	14	18.4	44	57.9	18	23.7
**Tiredness**	62	13.3	26	41.9	24	38.7	12	19.4
**Dysuria**	54	11.6	21	38.9	24	44.4	9	16.7
**Shortness of breath**	43	9.2	16	37.2	22	51.2	5	11.6
**Varicose veins**	35	7.5	15	42.9	19	54.3	1	2.9
**Swollen legs**	33	7.1	15	45.5	15	45.5	3	9.1
**Vaginal bleedings**	31	6.7	6	19.4	6	19.4	19	61.3
**Fever**	26	5.6	4	15.4	12	46.2	10	38.5
**Vaginal discharge/itching**	15	3.2	9	60.0	6	40.0	0	0.0
**Other**	46	9.9	11	23.9	21	45.7	14	30.4

Of the total number of 421 women that reported episodes of acute ill health, 356 (83.8%) reported that the ill health conditions affected their day-to-day work. Severe limitation of day-to-day work or total incapacitation was experienced by 282(60.5%) pregnant women. Work limitation was highest during bleeding episodes (61.3%). Fever (38.5%) and NVP (32.0%) were the other two conditions that had a notable effect on daily activities (Table 2).

Of the 421 pregnant women reported episodes of acute ill health, 260 (61.8%) women sought medical treatment. The total number of episodes that were treated by medical services treatment was 373. Hospitalizations were reported by 83 (17.8%) pregnant women and the total number of hospitalizations was 109. Total number of days spent in the hospital by the study sample for these conditions was 462, with an average of 4.2 days per admission. The leading cause of hospitalization was NVP accounting for 43.1% of total admissions and 49.1% of total days spent in hospitals (Table 3).

**Table 3:** Treatment seeking and hospitalizations for symptoms of acute ill health among 466 pregnant women in gestational age 24–36 weeks in Anuradhapura, Sri Lanka.

Symptom	Sought medical care (N = 466)^1^		Admitted to hospital (N = 466)^1^		Number of days in hospital (Total-462)^2^	
	n	%	n	%	n	%
**Nausea and vomiting**	95	20.4	47	10.1	227	49.1
**Heartburn/regurgitation**	46	9.9	2	0.4	2	0.4
**Cough and cold**	35	7.5	2	0.4	4	0.9
**Backache**	26	5.6	8	1.7	16	3.5
**Headache**	24	5.2	6	1.3	71	15.4
**Pain in pelvic area**	22	4.7	10	2.1	24	5.2
**Fever**	21	4.5	1	0.2	2	0.4
**Vaginal bleedings**	18	3.9	14	3	27	5.8
**Dysuria**	16	3.4	5	1.1	11	2.4
**Cramps**	14	3	0	0	0	0
**Dizziness**	11	2.4	1	0.2	1	0.2
**Shortness of breath**	9	1.9	3	0.6	9	1.9
**Tiredness**	5	1.1	1	0.2	25	5.4
**Varicose veins**	5	1.1	0	0	0	0
**Vaginal discharge/itching**	4	0.9	0	0	0	0
**Swollen legs**	3	0.6	0	0	0	0
**Other conditions**	19	4.1	9	1.9	43	9.3

1 Percentage was calculated out of total study sample.

2 Percentage was calculated out of total number of hospital days.

We analyzed occurrence of severe work limiting episodes of ill health (denoted by 0 and 1 on the visual analog scale) and hospital admissions by socio-demographic characteristics (Table 4). Although the total numbers were low, pregnant women belonging to Moor and Tamil ethnic groups reported more episodes of severe ill health compared to Sinhalese women. None of the socio-demographic characteristics were shown to be associated with these episodes of ill health during the antenatal period in these populations.

**Table 4:** Distribution of pregnant women who reported severe acute episodes of ill health by socio-demographic characteristics, among 466 pregnant women in gestational age 24–36 weeks in Anuradhapura, Sri Lanka.

	Episodes of severe ill health*					Significance (p value)**	
	Yes			No			
	n	%	n		%		
**Age**							
<20 years	19	52.8	17		47.2	.778	
20–35 years	207	53.6	179		46.4		
>35 years	26	59.1	18		40.9		
**Ethnicity**							
Sinhalese	230	52.6	207		47.4	.015	
Tamil^1^	3	100.0	0		0.0		
Moor^1^	19	73.1	7		26.9		
**Gestational age**							
24–28 weeks	80	51.0	77		49.0	.335	
29–36 weeks	172	55.7	137		44.3		
**Parity**							
1	106	55.5	85		44.5	.876	
2	75	53.2	66		46.8		
3 or more	71	53.0	63		47.0		
**Highest level of education**							
Up to grade 5	12	70.6	5		29.4	.353	
Up to grade 10	111	52.4	101		47.6		
Passed O/L	66	51.6	62		48.4		
Passed A/L	51	61.4	32		38.6		
University education	12	50.0	12		50.0		

* severe ill health was defined as those episodes that needed hospital admissions and severe work limitations as indicated by 0 or 1 in visual analog scale).

** based on Pearson Chi-square test.

1 these categories were amalgamated for chi-square testing.

## Discussion

Global evolution of maternal health services has been reinforced over the past few decades through evidence-based concepts as described in detail in numerous primary health guidelines and organizations including Primary Health Care (1978), Safe Motherhood (1987), and Reproductive Health (1994). There has been significant success in provision of interventions aimed towards modification towards human development and improvement of disease burden and productivity. The present paper discusses the prevalence of self reported common minor maternal ailments in Anuradhapura district in Sri Lanka and their impact on household productivity, thereby providing evidence to support further public health efforts aimed to reduce disease burden and loss in productivity.

The overall percentage of pregnant mothers having self-reported maternal ill health conditions was 90.3%. Although this value is more or less similar to the prevalence reported in community based studies in the South East Asian Region [10], [11], [12], the individual contribution of symptoms of morbidity is somewhat different in our study population. Common symptoms reported by the study participants in our study were nausea and vomiting (69.7%), back ache (32%), dizziness (24%) and heartburn/regurgitation (23%). A large scale community based study conducted in Bangladesh by Choudhury and colleagues [10], reported symptomatic anemia, palpitations, urinary problems and headache as the most prevalent symptoms during pregnancy. In contrast, symptoms of anemia were very low in our population and our studies showed that the prevalence of severe anemia was less than 0.5% and mild to moderate anemia was around 14% in the study area (Manuscript accepted for publication). It is worth noting that fever>3 days and urinary symptoms which were common in other populations in neighboring countries [10], [11], [12] were not common in this Sri Lankan population. One reason for this could be the comparatively higher levels of socio-developmental factors in this sample, such as high maternal education and better hygiene and sanitation that may have influenced the occurrence of infection during pregnancy.

Altogether 83.6% of pregnant women had disturbances to their daily activities due to these ill health conditions. Out of the four most prevalent conditions, nausea and vomiting during pregnancy was causing the highest incidence of severe or total incapacitation of pregnant women during the ill period (32%), accounting for 43.1% of hospitalizations and 49.1% total hospital days reported. NVP has been a major antenatal morbidity condition reported worldwide. Prevalence estimates depends on various factors. It has known effect on health related quality of life [13], [14]. It is correlated with maternal anxiety and depression [15], social support and psychological status of the pregnant women [16], [17] and also have created a considerable cost to families [18], [19]. Despite the known burden of this condition, available treatment options remain inadequately studied. [20] While use of antiemetic drugs has been controversial due to their suboptimal effectiveness as well as fear of possible side effects to the unborn child there is greater acceptance towards prevention of NVP episodes through behavioral and life style modifications. Similarly, simple lifestyle modifications coupled with common low-risk medications could also be applied to prevent other common symptoms such as gastroesophagael reflux/heartburn, back ache, varicose veins and leg edema [21].

Current healthcare practice is influenced by various sociocultural determinants. Over the course of our focus group discussion, common themes of medical practitioners treatment advice emerged. There seems a tendency of practitioners in this cultural context to merely to reassure pregnant woman that these ill health conditions are transient and avoidance of even simple medication is best. We propose that counter to this practice, there is immense possibility and capacity of promoting the mentioned healthy behaviours among pregnant women. Sri Lanka benefits from a strong public health system that promotes routine primary care with public health personnel throughout the country. Further improvements in antenatal morbidity from minor ailments might be obtained through educating both pregnant woman and clinic healthcare staff about accessible, effective and economical treatment options beyond reassurance. It would be highly cost effective to transition the current attitudes about highly prevalent but treatable conditions towards more active management. This will require development of proper preventive guidelines, aligned with accepted clinical practice, as well as creation of locally applicable management guidelines. Future evaluation of modern guideline implementation and outcomes should be pursued in a locally acceptable manner.

In contrast, vaginal bleeding in pregnancy is well recognized as a danger sign in the community requiring intervention. Women suffering this symptom are urged to seek healthcare assessment and typically then follow a regime of strict bed rest. Presence of vaginal bleeding during antenatal period though not common (6.7%), was the highest cause specific reason for total incapacitation of daily activities (61.3%) relative to incidence reported. This recognition and treatment reflects the underlying motivation present to provide high level of care to pregnant woman for medically treatable conditions. This is reflective of the socio-cultural attitudes of social protection and acceptance of “pregnancy” in Sri Lanka.

In contrast to other developing countries, an association of maternal morbidities with socioeconomic factors was not observed in this study population. Compared to the mentioned characteristics of the populations studied elsewhere, this study population has minimum amount of social disparities. The level of education of pregnant women in this population is much higher than other developing countries. These factors together with equitable distribution of public health services and cultural factors such as minimum gender inequalities may have contributed to the lack of disparity across socioeconomic divisions in society.

Studying the etiology, associations and consequences of minor acute ill health conditions are often neglected within developing countries. Due in part to the paucity of research, there is lack of evidence for recommendations to prevent and overcome these conditions. [20], [22], [5] [23]. The lack of sufficient research could be due to problems in interest, prioritization and also funding. As we have shown in this study, the minor maternal ill health conditions impact day-to-day life and have a significant burden on women during pregnancy. Evidence based management guidelines and health promotion strategies are needed to control and prevent these conditions, in order to provide quality and comprehensive maternal health care aimed on improved maternal, child and, indeed, societal development outcomes.

### Limitations

Interpretation of evidence from our study should be done in context of its limitations. As a cross sectional study, we are unable to accurately account for incidence of all episodes of illness during pregnancy. Two major limitations are could affect the morbidity estimate in this study. Selection of currently pregnant women introduced a significant selection bias to our sample by excluding woman who experienced miscarriages. Furthermore, pregnant women who were hospitalized or having severe limitation of activities for a long duration during the data collection period of the study would have had a probability of not to be included in the study. These biases would have contributed to the observation of “minor ailments” as leading causes of morbidities and an underestimation of actual burden of morbidities. In addition, for mothers with a gestational age of 6–8 months, the recall period may have been too long. We used frequency of treatment seeking and hospitalization, validated through clinic books, diagnosis cards and the pregnancy records to minimize this effect of recall bias. Prior population based surveys have been proved to provide valid estimates of maternal morbidities. Therefore, we would like to interpret these observed morbid conditions without commenting on known major maternal morbidities that would be better evaluated by a cohort study.
